# A pragmatic approach for implementation of value-based healthcare in Amsterdam UMC, the Netherlands

**DOI:** 10.1186/s12913-022-07919-1

**Published:** 2022-04-25

**Authors:** Florence A. C. J. Heijsters, Fenna G. F. van Breda, Femke van Nassau, Marije K. J. van der Steen, Piet M. ter Wee, Margriet G. Mullender, Martine C. de Bruijne

**Affiliations:** 1 Department of Plastic, Reconstructive and Hand Surgery, Amsterdam UMC, Vrije Universiteit Amsterdam, Boelelaan, 1117 Amsterdam, Netherlands; 2 Department of Strategy and Innovation, Amsterdam UMC, Vrije Universiteit Amsterdam, Boelelaan, 1117 Amsterdam, Netherlands; 3 Department of Nephrology, Amsterdam UMC, Vrije Universiteit Amsterdam, Boelelaan, 1117 Amsterdam, Netherlands; 4 Department of Public and Occupational Health, Amsterdam UMC, Vrije Universiteit Amsterdam, Amsterdam Public Health Research Institute, Boelelaan, 1117 Amsterdam, Netherlands

**Keywords:** Healthcare quality improvement, Patient-centred care, Quality improvement methodologies, Transitions in care, Teamwork, Value-based healthcare

## Abstract

**Background:**

The emphasis on implementation of value-based healthcare (VBHC) has increased in the Dutch healthcare system. Yet, the translation of the theoretical principles of VBHC towards actual implementation in daily practice has been rarely described. Our aim is to present a pragmatic step-by-step approach for VBHC implementation, developed and applied in Amsterdam UMC, to share our key elements. The approach may inspire others and can be used as a template for implementing VBHC principles in other hospitals.

**Methods:**

The local approach is developed in a major academic hospital in the Netherlands, based at two locations with 15,000 employees in total. Experience-based co-design is used, building on our learning experiences from implementing VBHC for 14 specific patient groups. The described steps and activities devolved from iterative and participative co-design sessions with various experienced stakeholders involved in the implementation of one or more VBHC pathways.

**Results:**

The approach includes five phases; preparation, design (team introduction, outcome selection, action agenda), building (outcome set integration in daily practice), implementation (training, outcome registration and implementation) and the continuous improvement cycle. We described two cases for illustration of the approach; the Cleft Lip and Palate and the Chronic Kidney Disease patient groups. For a good start, involvement of a clinical leader as driving force, ensuring participation of patient representatives and sufficient resources are needed.

**Conclusion:**

We have experienced that several defining features of the development and implementation of this approach may have contributed to its completeness and applicability. Key elements for success have been organisational readiness and clinical leadership. In conclusion, the approach has provided a first step towards VBHC in our hospital. Further research is needed for evaluation of its effectiveness including impact on value for patients.

**Supplementary Information:**

The online version contains supplementary material available at 10.1186/s12913-022-07919-1.

## Background

Worldwide, healthcare systems face the challenge of increasing costs [1]**.** In 2006, Porter and Teisberg introduced the principles of Value-Based Health Care (VBHC). These principles are intended to reduce costs in healthcare, while at the same time increasing the quality of healthcare by increasing the value for patients [2]. In 2013, Porter and Lee presented a strategic vision to implement VBHC. This vision includes identifying disease specific patient groups and developing a multidisciplinary team that is focused on patient centred care for this group [3–5]. Measuring clinical and patient reported outcomes (PROs) is an essential element in this VBHC process. To standardize outcome measurement within specific patient groups, the International Consortium for Health Outcome Measurement (ICHOM) develops standard minimum sets of patient reported outcome measures (PROMs). The aim of monitoring outcomes is to improve the value of healthcare by enabling comparison of these outcomes at the levels of the individual patient and patient groups [6–8]. Analysis of these data enables healthcare professionals to validate choices, guide improvements, learn from colleagues or other hospitals, and to motivate themselves or the team to collaborate or make changes. Ultimately, optimization of care for disease specific patient groups with regard to the quality of care and cost reduction is strived for to ensure that society is provided with affordable and accessible care **[**9**]**.

Despite the overwhelming popularity of VBHC, there is also ambiguity about the interpretation and operationalization of the VBHC concept [10–12]. Steinmann et al. showed that the very meaning of VBHC is subject to interpretation. Their research revealed different perceptions regarding VBHC, ranging from the conviction that VBHC is a toolkit to incentivize providers to the assumption that VBHC is ‘a dogma of manufacturability’. The use of PROMs and comparison of outcomes both regularly meet with resistance and scepticism from clinicians [13–15].

### Implementation of VBHC is challenging

The path for transforming current health care towards value-based healthcare is challenging, because it entails both a new collaborative relation between healthcare professionals and patients, as well as fundamental change from an organisation structured around medical specialisation towards an organisation centred around a multidisciplinary care pathways for patient groups. This transformation may be regarded as a complex socio-technical intervention that requires organisational readiness for change among healthcare professionals, including cultural change [16–19]. As one of the founders of VBHC stated: *“No one should expect the value framework to be easy to implement. The measurement of outcomes and costs, the organisation of clinicians into teams focused on improving care for patient populations ( …*) *are all formidable tasks”* [16].

The emphasis on VBHC has also increased in the Dutch healthcare system, in line with the widespread international uptake of VBHC. Vision and mission statements of formal institutions, such as the Dutch Healthcare Institute and Netherlands Federation of University Medical Centres (NFU), mention VBHC as a key element with a focus on improving outcomes in daily practice that matter to patients or populations while optimizing resource utilization [6–8]. Several initiatives related to implementation of VBHC have been developed to share knowledge about VBHC between healthcare professionals. Santeon, a collaboration between several non-academic teaching hospitals in the Netherlands, started as forerunner with benchmarking by developing scorecards with clinical outcomes, cost and process metrics [20]. Subsequently, the NFU started a collaboration with regard to VBHC. Because of the mostly tertiary care, they are focusing on regionally integrated care, rare diseases and an advanced training program for doctors, nurses, data scientists and implementation support staff [6]. In addition, the Linnean Initiative initiated an open network across disciplines and domains, and the Ministry of Health, Welfare and Sport has recently set up an action program focusing on outcome-based care [21, 22].

As VBHC is such a complex intervention, many start with implementing only one aspect that seems most feasible for them at the time [23, 24]. In practice, each hospital has its own way and preference for translating the VBHC principles into practice, on the basis of what is possible at the hospital concerned, and the healthcare system [25, 26].

### From theory to practice

Porter et al. elaborated on various important principles of VBHC, but did not specify *how* these principles may be applied in practice. The translation of the theoretical principles of VBHC towards actual implementation in daily practice have rarely been described [3, 16, 27]. Garvelink et al. state that there is a need for a pragmatic approach, which can be scientifically evaluated [28].

Hence, this study aims to describe a pragmatic step-by-step approach focusing on the process to get started with the implementation of VBHC in practice, applied in a tertiary centre. In this article, we illustrate the implementation process by two practical examples of multidisciplinary care pathways in Amsterdam UMC, the Netherlands.

## Context and setting

In the Dutch Healthcare system, (basic) medical insurance is compulsory for all citizens and care is delivered by private and public health care providers, who have agreements with health insurance companies. There are 69 hospital organisations in the Netherlands, including 8 university medical centres. Amsterdam University Medical Centre (Amsterdam UMC) is a major academic hospital, with two locations and 15,000 employees in total. The board of directors is responsible for its management and strategy, which is carried out in a line organisation, comprising 10 divisions and several staff services divided over two locations; the Amsterdam Medical Centre (AMC) and VU University medical centre (VUmc). Enhancing patient value by delivering personalized care throughout the full cycle of care is one of the main strategic pillars of the Amsterdam UMC strategy.

### VBHC in Amsterdam UMC

The principles of VBHC were introduced in Amsterdam UMC by the hospital’s Strategy and Innovation (S&I) department in 2017, inspired by the Karolinska Institute’s strategy and organisation of care in Sweden. The purpose of introducing the VBHC principles in Amsterdam UMC was to improve quality of care for multidisciplinary patient groups, based on clinical, patient-reported and process outcomes by strengthening the process of continuous quality improvement using VBHC principles. Amsterdam UMC first started with the implementation of a continuous improvement cycle with regard to the process of care and health outcomes [3].

From an organisational point of view, the overall goal was to learn from each patient to make future care more effective and efficient. These goals were translated into a local VBHC program, commissioned by the board of directors. The organisation and implementation of this program was delegated to the department of S&I.

## Organisation and development of approach

### Program organisation

**Fig. 1:**
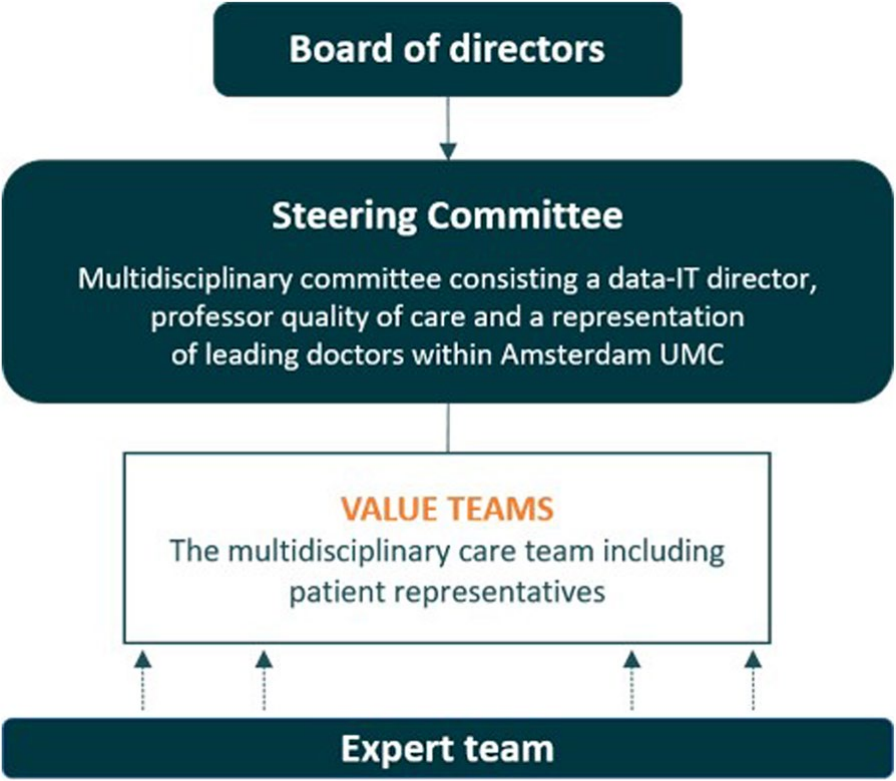
Program organisation

From the start, the VBHC program in Amsterdam UMC has been directed by a steering committee chaired by the chief medical officer (Fig. 1). The steering committee has determined the program-wide goals, monitored progress and selected new teams who wanted to start with VBHC.

*Value teams* executed the VBHC approach. A value team in Amsterdam UMC served as an Integrated Practice Unit (IPU), which means a multidisciplinary care team around a specific patient group, comprising staff and patients who are involved in this care pathway [29]. The value teams were led by a clinical lead; this was a physician or nurse working as a representative of the multidisciplinary care team.

One general expert team guided the value teams. This team was formed by consultants from the S&I department, data- and IT experts and various experts for specific themes (e.g. clinimetrics, PROMs, shared-decision making or costs). They guided the different value teams with procedural experience and knowledge, during the implementation of VBHC. The expert team has evolved over time. At first, an external consultant, experienced with implementing VBHC in other Dutch hospitals, was employed to ensure a quick and adequate start of VBHC implementation in Amsterdam UMC. This consultant was engaged in the first 4 value teams. Value teams were guided by the clinical lead and supported by the external consultant together with an internal consultant. These first implementation cycles were seen as the pilot phase of the VBHC-program. Based on the experiences in the pilot phase, a structured implementation approach was established. With this, internal consultants facilitated the value teams to implement VBHC. Currently, 14 teams in Amsterdam UMC were working on, or preparing for, the implementation of VBHC.

### Experience-based co-design

The method for implementing VBHC in Amsterdam UMC is based on “experience-based co-design” (EBCD). This design combines a user-centred orientation (“experience-based”) and collaborative change processes (“co-design”) to identify and co-design improvements [30]. This approach is especially suitable to achieve service-design for patients [31–34]. Our focus for using this design is on achieving 1) personalized care, based on the wishes and needs of each individual patient, 2) efficient care, by enhancing the care process, 3) optimal teamwork between multidisciplinary professionals and the patient, and lastly 4) insight in outcomes to ultimately improve the patient value of healthcare.

### Process of describing the VBHC approach

The lead author (F.H.) was one of the expert team members and followed the process during implementation for the last 3 years. To describe the step-by-step approach, data was collected from multiple sources alongside the co-design and implementation process, and made available by the clinical leaders (F.vB., P.DG.) and a team member (M.vdS.) who was involved in both cases. We collected documents and field notes from the workshop meetings during the design phases and related meetings of the value team throughout the implementation. In addition, existing qualitative semi-structured interviews to assess facilitators and barriers for implementation with the clinical leads of the CLP and CKD teams were used [35]. Finally, the described approach devolved from iterative sessions with various experienced stakeholders involved in the implementation of one or more VBHC pathways: expert team consultants and the authors, including senior researchers, the program manager and the chief medical officer. The development of this approach in iterative sessions resulted in a highly robust and structured step-by-step approach in 2020, but will be in ongoing improvement in the future.

### Patient and public involvement

Patients are members of the value team. This ensures the inclusion of wishes and needs of the patient group in the enhancement of the care process and that relevant outcomes are selected to measure in a later stage. The design of this approach is based on learning experiences from value teams, including the workshop meetings where patients were involved.

### Illustrating pilot cases

To illustrate the described methodology for implementing VBHC in current clinical practice in Amsterdam UMC we described two cases: the multidisciplinary Cleft Lip and Palate (CLP) team and Chronic Kidney Disease (CKD) team, see Table 1. The selection was based on their difference in medical context, starting moment with the VBHC implementation, and thus related expert team.

**Table 1:** Characteristics of the two cases

Cases	Cleft Lip and Palate	Chronic Kidney Disease
*Patient inclusion criteria*	Patients with a cleft lip and/or palate.	Patients with a eGFR of ≤20 ml/min and not yet started with renal replacement therapy
*Total time of care pathway*	Starting at a prenatal age until the age of 22 years	Varying from several months to several years
*Multidisciplinary value team members*	• Plastic surgeon • Ear, nose, throat specialist • Oral and maxillofacial surgeon • Speech therapist/pathologist • Cleft care nurse (specialist) • Paediatric dentist • Orthodontist • Paediatrician • Geneticist • Psychologist • Social worker	• Internist-nephrologist • Nurse (specialist) • Vascular surgeon • Geriatrician • Dietician • Social workers • Managers • Back office and outpatient clinic staff • Two patient representatives
*Organisation of care*	Tertiary care	Integrated care (primary, secondary and tertiary care)
*Start with VBHC in*	May 2017	December 2017
*Guided by*	Expert team including external and internal consultant	Expert team including internal consultant
*Outcome set*	ICHOM Cleft Lip and Palate [36]	ICHOM Chronic Kidney Disease [37]

The CLP team started with implementing VBHC in 2017 as one of the pilot teams. Cleft lip and/or palate is a congenital anomaly with different manifestations. Cleft care is complex and prolonged, starting at a prenatal age until the age of 22 years. Due to its complexity, various medical disciplines are involved during the care pathway. Simultaneously with participation in the VBHC program, two formerly local CLP teams based at the two locations of Amsterdam UMC were merged into one team.

The second case, the multidisciplinary CKD team, started at the end of 2017 with implementing VBHC principles for patients with a glomerular filtration rate (eGFR) of 20 ml/min or lower that had not yet started with renal replacement therapy (RRT). These patients visit the nephrology outpatient clinic in order to slow down the progression of kidney failure by medication or diet. This outpatient clinic is organised in such a way that patients can see several care professionals from different disciplines during one visit. At the same time these patients are being educated about kidney transplantation, dialysis or conservative therapy. Structural recording of outcomes that matter to patients was missing at this outpatient clinic. To learn whether they are offering the right care to this complex group of patients was one of the reasons to start with VBHC [38].

## Step by step approach towards value-based healthcare

**Fig. 2:**
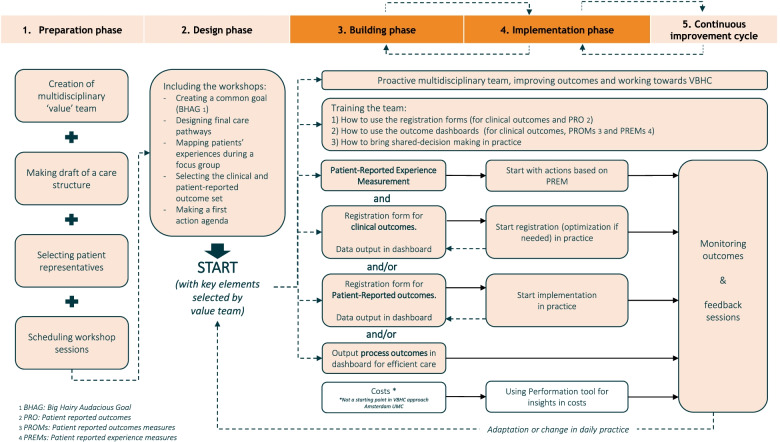
VBHC approach for a specific patient group by phased activities

The experiences and lessons learned of multiple teams have resulted in this pragmatic and iterative approach that includes five phases as shown in Fig. 2. All value teams follow the same steps, tailored to the dynamics and needs of their patient group. This provided a solid foundation for the start of the implementation of VBHC in Amsterdam UMC.

A detailed description of the approach, divided into phases with their goals, tasks and tools, is specified in Table 2. By illustrating two cases (CLP/CKD) of the value teams and the performed activities and deliverables during VBHC implementation, we provide insight into how this approach developed. Both cases have largely followed the same steps. A detailed description including activities, deliverables, facilitators and challenges during the implementation of our approach is provided in Additional file 1, where the main differences are highlighted. The following described preconditions and key points are based on the experiences of our pilot cases (Additional file 1).

**Table 2:** The description of each phase with their goal, tasks and support

		Goal	Tasks	Support	
				What?	Who?
**1**	**Preparation phase**	• Ready to start with the VBHC design phase with a multidisciplinary team including patient representatives	• Select a specific patient population and delineate it • Create a multidisciplinary value team around a specific patient group • Make a draft care structure • Select patient representatives who can participate in the design sessions • Schedule sessions (5) for preparation design phase	1. Coordination of tasks	1. Internal consultant of VBHC expert team, together with clinical lead
**2**	**Design phase**	• Common short- and long-term ambition of VBHC with the multidisciplinary value team • Patient representatives are involved in team and focus group are conducted for expressing their wishes and needs • An established plan for how the outcomes set will be used in the clinical care process • Jointly defined key elements and actions of VBHC for the specific patient group to start improving the value of care for the specific patient group	• Use a BHAG for forming a common goal • Design the final care pathways • Map the patients’ experiences with the care process and their wishes and needs • Select an appropriate clinical and patient-reported outcome set • Ensure an action agenda; existing of short- and long term actions that will improve the value of care	1. Facilitation of workshop sessions (5) 2. Moderation of patient focus group Tools • Format BHAG • Format final care pathway • Format questions focus group • Format action agenda • PREM dashboard	1. Internal consultant(s), optionally with consultant of PROM expertise point 2. Experienced moderator
**3**	**Building phase**	• Proactive multidisciplinary value team working with defined actions • The outcome set is ready to be used in practice, so that they can be filled in for & by every patient • New registration rules are embedded in the care process • The selected outcomes for the patient and the care givers are visualised in dashboards	• Integrate the outcome set in the electronic health record by the principles of unambiguous documentation • Ensure a uniform and clear registration process by multidisciplinary team • Build an improvement dashboard at population level • Build a dashboard for improving personalised care at patient level • Select process outcomes to use for continuous improvement daily care process	1. Advice on process 2. Advice on content and outcome selection 3. Support for building clinical outcome set in the electronic health record and for visualizing PROMS in a dashboard at patient level 4. Support for visualising data in improvement dashboard 5. Training for using PREM dashboard in practice	1. Internal consultant of VBHC expert team 2. Consultant of PROM expertise point 3. Data-IT expert of EHR department 4. Data-IT expert of Business Intelligence department 5. Clinical lead together with internal consultant of PREM team
**4**	**Implementation in daily practice**	• The implementation of various elements of value-based working have been incorporated in the care process of the specific patient group • Proactive multidisciplinary team, improving outcomes and working towards VBHC • Shared decision-making in the consultation room	• Formulate improvement actions based on PREM results • Train the healthcare team how to use the outcome registration forms in practice • Prepare the healthcare team for shared decision-making in practice through training	1. Training how to use the outcome registration forms in practice 2. Training shared decision-making by PROM expertise point 3. Training for using the dashboard, at patient level, in practice	1. Consultant of PROM expertise point 2. Consultant of PROM expertise point 3. Consultant of EHR department and PROM expertise point
**5**	**Continuous improvement cycle**	• Continuous improvement of care by multidisciplinary value team • Follow-up steps towards value-driven working are determined	• Monitor the implementation process and improvements in daily practice • Organise feedback sessions with multidisciplinary value team	1. Training for using the population dashboard in practice 2. Feedback sessions to discuss actions, results and the implementation process	1. Data-IT expert / internal consultant 2. Clinical lead (option: together with internal consultant)

*VBHC* Value based healthcare, *BHAG* Big Hairy Audacious Goal, *PRO* Patient reported outcomes, *PROMs* Patient reported outcomes measures, *PREMs* Patient reported experience measures

In order to give the team a good start and to ensure organisational readiness, both from the support services and the department of the proposed ‘value’ team, we define the following certain preconditions: First, select an appropriate *multidisciplinary care pathway *around a specific patient group. To meet this requirement, the team must have improvement goals for this patient group that match the VBHC principles. These goals may relate to different elements of the VBHC approach. For example, the CKD team mainly focus on offering the right care for our patients and the CLP team on redesign of care, including outcome measurement.

Second, it is necessary to involve an enthusiastic *clinical leader as driving force* on behalf of the multidisciplinary team. This clinical lead must be a healthcare professional with enough time, motivation and resources to support this process. In both of our cases, this was a medical specialist.

Subsequently, *patient representatives* should be available to participate with the multidisciplinary value team during the design and implementation process. Each team did this in its own way. The CLP team involved patients in various ways (questionnaires or during workshops). In contrast, the CKD team ensured structural patient participation during the design phase and then on by hiring a patient to continuously give feedback on how to improve the delivery of care.

Lastly, *resources need to be available* both from the support services and the healthcare department itself. In addition, departments’ willingness to implement changes in their work process need to be present. These are important parts to ensure organisational readiness [17]. When these preconditions are met, the step-by-step approach can be applied.

A team can indicate that they wish to start with VBHC via an intake form. When the steering committee approves this intake, by matching their goals and mutual expectations with regard to motivation, the team starts. In this approach, each phase has its own goal and key points with associated activities and stakeholders (see Fig. 2 and Table 1). First key point, to complete the preparation phase, the value team including patient representatives, must be formed to start the design sessions. In the design phase, the value team works together in several sessions, together with PROM-experts they select an outcome set and jointly establish short and long-term goals and actions for their VBHC implementation, appropriated to the needs of the patient group. A subsequently key point; the team makes a plan on how to use the outcome set in the clinical care process. In addition, in this phase, time is also made for what the team needs, such as the attention to teambuilding in the CKD team.

When the building phase starts depends on the capacity of data-IT experts. Therefore, the duration of this phase can vary between teams. After the design sessions, the proactive multidisciplinary value team immediately starts working on a regular basis with the defined actions and already available outcomes, for example the PREMs (patient-reported experience measures) or process outcomes. This was not yet applied during the pilot phase, but was taken into account as a lesson learned for further deployment of this VBHC approach, thus also in the CKD team.

As soon as the clinical and patient-reported outcomes are built in the EHR-system, the implementation phase starts after training the healthcare professionals to apply it in practice. To accomplish the building phase, the registration of the outcome set is embedded in the care process and the IT department delivers a dashboard for the value team where the outcomes are visualised.

The final two phases, implementation and continuous improvement, regularly overlap each other. The implementation of various elements of VBHC are incorporated in the care process of the specific patient group. As part of this, the healthcare professionals receive training for shared-decision making in the consultation room and the value team monitors regularly the outcomes together. Feedback is taken into account during the implementation again.

## Reflection and recommendations

We have experienced that several defining features of the development and implementation of this approach may have contributed to its completeness and applicability. These were categorized into five elements, which will be discussed below; organisational readiness, clinical leadership, experience-based co-design (EBCD), role of expert team and monitoring outcomes. All elements are important on their own, but for a successful VBCH approach, their combination has proved essential.

Organisational readiness is a shared psychological state in which organisational members feel committed to implementing an organisational change and confident in their collective abilities to do so [17]. Within our VBHC approach, the importance of this concept became apparent as “organisational readiness” developed only over time, which manifested in later teams progressing more efficiently than the earlier teams. The lack of organisational readiness at the start of the VBHC approach in Amsterdam UMC was partly due to the bottom-up approach by conducting a pilot among enthusiastic pioneer value teams. This implied that there was team readiness, but organisational readiness was still deficient, resulting in a lack of capacity for data/IT support on the organisation level. In our cases, the dynamics in our organisation (e.g. merger) was a challenging factor which also effected and delayed the organisational readiness. Therefore, we recommend for future implementation to have sufficient support from the Board of Directors and line organisation of the VBHC program before starting, to embed the program well in the organisation [39]. Capacity for data/IT support must be prioritised by them and the supporting departments in advance, to accomplish the building phase, otherwise the implementation process will be delayed significantly which in turn causes demotivation of the team.

As stated before, to implement VBHC in a “traditionally organised” hospital, fundamental changes in daily practice are needed which require clinical leadership. Within this approach, the clinical lead, who heads the value team, serves as the primary clinical leader. Several factors were found to contribute to the success of this role. First, the leader needs to have the skills to bring about change, which include enthusiasm, good advocacy skills, effective communication and creating ownership together with the team. Second, a clinical lead with mandate and authority is needed, endorsed by the clinical team and their departments [40]. Our advice, therefore, is to provide an enthusiastic clinical leader with authority as a driving force on behalf of the multidisciplinary team. This clinical leader should be a healthcare professional with sufficient time, motivation, mandate and resources to support this process and to ensure that the ideology and ownership is carried through the department.

Experience-based co-design has, as mentioned earlier, a user-centred orientation in combination with collaborative change [30]. We did not include all eight stages of experience-based co-design, but extracted the most important ones that fitted our goal and approach [34]. The fact that with the same approach, different solutions, actions and outcomes have been formulated for different value teams, shows that the approach is a useful blueprint for a variety of different multidisciplinary healthcare teams, i.e. patient populations and stages of transformation. Diverse strategies evolved due to the distinct experiences of the stakeholders involved, including the patient representatives. It also became clear that each patient group has its own wishes and needs, which could be taken into account by using this co-design. These involved stakeholders are necessary during the redesign of the care process [39].

The expert team played a central role in our approach. They ensured connection between value teams, the supporting and facilitating departments (data/IT, communication, etc.) and the steering committee. The involvement and level of guidance of the value teams varied in our cases. We have learned that the autonomy of value teams had a positive impact on ownership by the team to ultimately ensure improvement in quality of care. When getting started with VBHC, make sure the value team is not pampered by central support. This may prevent lack of capacity and ownership. When scaling up and implement VBHC, the value team must take ownership for itself. Thus, the role of the expert team is to pay attention to ownership from the start and help the healthcare teams do the implementation themselves in which the consultants are primarily involved as experts rather than executors. To ensure knowledge sharing and sustainable employability of the experts, the expert team initiated a PROM expertise point within Amsterdam UMC and developed a VBHC toolbox. With the toolbox the value teams can get started themselves by choosing a focusing element for their start with the implementation VBHC, so that it becomes a little more manageable.

Lastly, it is important during implementation to monitor progress towards implementation goals [41]. Our experiences in the CKD and CLP value teams showed that getting straight to work with existing available outcomes was a facilitator for team motivation and subsequent steps towards implementation. As an example, in addition to long-term outcomes (e.g. 5-year survival), they formulated also short-term outcomes (e.g. surgical complications, visits emergency department) and used existing PREMs. The team defined improvement goals for these outcomes and monitored the goals. In addition, it will also be beneficial in the future if the hospital opts for one integrated system for the case mix, clinical and patient reported outcomes. Preferably linked to the hospital’s own EHR, so that the building phase can proceed quickly and the results can be collected and monitored in an unambiguous form.

## Discussion

Value-based healthcare was introduced in Amsterdam UMC in 2017. In this article, we describe a pragmatic approach for the implementation of VBHC among value teams in Amsterdam UMC. At present, 14 value teams are working on improving the quality of care for their patient groups by using this approach.

The approach described here serves as a first step for the implementation of VBHC, knowing that it only incorporates a number of key principles from Porter’s strategy [2]. We have chosen to simplify this originally complex process in our approach. At this point one can say the approach entails mainly a strategy for Patient Centred Care (PCC), as it lacks measurement of costs, which is an essential principle of VBHC [4, 5, 42]. At this moment, we mainly focus on improving the care process based on wishes and needs of the patients and on using outcomes at individual level in the consultation room including shared decision making, which both require culture and behavioural change [19]. This focus is a good starting point for future research into the effect of these outcomes (on an individual and population level) on quality improvement and ultimately the costs.

Strength of the present study includes the applied and tested approach which was developed over four pilot cases and refined during ten subsequent cases. As far as we know, we are the first who describe a detailed VBHC implementation with a learning approach based on evolving insight. Some limitations also need to be mentioned. First, team members of the VBHC expert team and two clinical leads were involved in development of the approach and writing process of this manuscript which may have limited the objectivity of our observations. However, this engagement also ensures valuable reflection. The impact of this close involvement of the clinical leads has been reduced by assessing the approach in iterative sessions with a wider delegation of stakeholders. Second, this approach, with associated expert team and infrastructure, will possibly not be applicable in every hospital, which reduces its potential for scalability. In addition, implementation often took a long time and therefore the effects of the outcomes and the activities of our approach are only visible at a later stage, but our advice to start immediately with the available outcomes will ensure an early start of the improvement cycle.

## Conclusion

In conclusion, this approach has provided a good starting point for implementation of VBHC in a tertiary hospital. The approach evolved over time, while simultaneously the implementation process improved. This blueprint provides guidance for the implementation of VBHC in daily practice, to ultimately ensure care that is of personal and measurable value for the individual patient. So far the focus of the implementation in Amsterdam UMC has been gaining insight in the clinical-, process- and patient reported outcomes. The description of this pragmatic approach intended to serve as an inspiration for others who want to start implementing VBHC. In the future, this approach and the implementation of VBHC on an individual and population level will be evaluated, in order to ultimately improve the quality of care in our hospital and nationally.

## Supplementary Information

**Additional file 1.** VBHC approach illustrated by the Cleft Lip and Palate and the Chronic Kidney Disease cases. The main differences are highlighted by using*.

## Data Availability

Not applicable.

## Abbreviations

Amsterdam UMC: Amsterdam University Medical Centres
BHAG: Big Hairy Audacious Goal
CKD: Chronic Kidney Disease
CLP: Cleft Lip and Palate
EBCD: Experience-Based Co-Design
eGFR: Glomerular Filtration Rate
EHR: Electronic Health Record
ICHOM: International Consortium for Health Outcome Measurement
IPU: Integrated Practice Unit
PCC: Patient Centred Care
PREMs: Patient Reported Experience Measures
PRO: Patient Reported Outcomes
PROMs: Patient Reported Outcomes Measures
VBHC: Value-based healthcare
