# Supplementary material for: A pragmatic approach for implementation of value-based healthcare in Amsterdam UMC, the Netherlands
Source: BMC Health Serv Res. 2022 Apr 25;22:550. doi: 10.1186/s12913-022-07919-1 (PMC9040233; doi:10.1186/s12913-022-07919-1)
Supplement: Supplementary file 1 — Additional file 1. VBHC approach illustrated by the Cleft Lip and Palate and the Chronic Kidney Disease cases. The main differences are highlighted by using*. [file 12913_2022_7919_MOESM1_ESM.pdf]

APPENDIX

Appendix 1: VBHC approach illustrated by the Cleft Lip and Palate value team (case 1) and the Chronic Kidney Disease value team (case 2).
The main differences are highlighted by using.* (*VBHC: Value based healthcare, PRO: Patient reported outcomes, PROMs: Patient reported outcomes measures, PREMs: Patient reported experience measures*)

<table>
<tr><th></th><th></th><th>Preparation phase</th><th>Design phase</th><th>Building phase</th><th>Implementation phase</th></tr>
<tr><td rowspan="3">Case 1

Cleft Lip and Palate</td><td>Description of activities and deliverables during approach</td>
<td>Activities:
• Meeting clinical lead and external and internal consultant VBHC expert team
• Schedule workshop sessions
• Selecting two patient representatives to attend workshop 3 *

Deliverables:
• Multidisciplinary ‘value’ team is composed including a clinical lead
• Draft version care structure is made
• Workshop sessions are scheduled</td>
<td>Activities:
• Workshop 1: Presentation about VBHC theory and designing a care structure for cleft lip and palate patients
• Workshop 2: Designing final care pathways + ranking clinical & patient-reported outcomes
• Workshop 3: Ranking the outcomes together with 2 patient representatives *
• Workshop 4: Selecting outcomes (clinical, PROs) and measurement tools (PROMs and PREMs)
• Workshop 5: Evaluation of pilot implementation of the new care pathway (and adjustments were made where necessary)

Deliverables:
• Qualitative patient experience surveys (n=20) as input for workshop 2 and 4 *
• Multidisciplinary CLP management team (MT) is set up to monitor and evaluate progress after workshop 5
• Clinical and patient-reported outcome set (including ICHOM)
• Collective mentality to get started with VBHC</td>
<td>Activities:
• Every 6 weeks MT meeting to monitor progress
• Every 4 months team meeting to discuss the progress and create new actions
• Multiple meetings with data/IT experts to design the outcome registration form including ‘patient label’ to recognize the population in the electronic patient record
• Multiple meetings with data analysts Business Intelligence (BI) to visualize population data output in dashboard

Deliverables:
• Registration form for clinical outcomes and case mix variables
• Embedded PROM tool including dashboard for the involved healthcare professionals</td>
<td>Activities:
• Team is trained how to use the clinical registration forms
• Team is trained how to use the PROM tool, patients are invited to fill in the PROMs
• Team is trained to bring shared-decision making in practice
• Using PROMs in clinical practice (discussed with patients during consultation multidisciplinary team)

Deliverables:
• Insight PROMs on patient-level which are discussed in team before consultation
• Insight population data in BI dashboard</td></tr>
<tr><td>Facilitators</td>
<td colspan="2">• Ranking the outcomes together with the value team provided a lot of insight
• Enthusiastic clinical lead who is intrinsically motivated to get started with VBHC
• Basic outcome set had already been established by ICHOM
• Having a discussion together in several sessions about why you want to start with VBHC and how you together want to organise care has a positive effect on the support.
• Inspiration from good examples in other hospitals</td>
<td colspan="2">• Periodic coordination and cooperation in the MT
• Support from the supporting departments has been essential for success.
• Knowledge sharing between value teams provided inspiration
• The value team felt supported by the board of directors.</td></tr>
<tr><td>Challenges</td>
<td colspan="2">• There was too little attention for personal introduction in the workshops
• Resistance was experienced to ‘re-create’ a care pathway together. As a result, the step to create a bundled care pathway together took a lot of time.
• Active patient participation was not a structural part of the workshops in the design phase.</td>
<td colspan="2">• The continuous involvement of the entire value team. The MT often made a decision, but it took a long time for the team to be informed. This results in the regular explanation and training of the team members in the registration and SDM process, despite the fact that you have done this training together.
• The building phase took a long time because there was not enough capacity from the EHR, BI and ICT departments.
• CLP value team was a forerunner in the merger, which meant that there were still different BI systems, which meant that a joint dashboard could not be set up for both locations. The choice was made to make a design on one system, but this resulted in less representative results and limited implementation.
• It took a long time before they could get started with the first outcomes.</td></tr>
</table>

		Preparation phase	Design phase	Building phase	Implementation phase
Case 2<br>*Chronic Kidney Disease*	*Description of activities and deliverables during approach*	**Activities**:<br>• Meeting clinical lead and internal consultant VBHC expert team for an explanation of the workshops and defining the specific patient group<br>• Schedule workshop sessions<br>• Selecting two patient representatives to attend all workshops *<br><br>**Deliverables**:<br>• Multidisciplinary 'value' team is composed including a clinical lead<br>• Draft version care structure is made<br>• Workshop sessions are scheduled	**Activities**:<br>• Workshop 1: 'Value based thinking'. Meeting the team through speed dates, presentation about VBHC theory and creating a common goal using BHAG (Big Hairy Audacious Goal).* The draft version of a care structure was presented and discussed.<br>• Workshop 2: Focus group with patient representatives (including 2 patient team members) about their experiences with the care process and their wishes and needs.* These insights were incorporated into the care pathway together with the team in a brown paper session.<br>• Workshop 3: Brainstorm and ranking relevant outcomes (clinical, PROMs and PREMs) and how to reduce costs.<br>• Workshop 4: Designing the final care pathway together. Also discussed inspirational examples of VBHC for the action/innovation agenda for short- and long term.<br>• Workshop 5: Presenting the final outcome set with clinical- and process outcomes, PROMs and PREMs. Choices for short-term actions were made. * Roles and responsibilities are determined.<br><br>**Deliverables**:<br>• Multidisciplinary CKD management team (MT) is set up to monitor and evaluate progress<br>• Clinical and patient-reported outcome set (including ICHOM)<br>• Existed PREM results are used for action agenda *<br>• Ensure an action agenda; existing of short- and long term actions that will improve the value of care *	**Activities**:<br>• Every 6 weeks MT meeting to monitor progress<br>• Every 6 months value team meeting to discuss the progress and create new actions<br>• Team is trained to bring shared-decision making in practice<br>• Multiple meetings with data/IT experts to design the outcome registration form including 'patient label' to recognize the population in the electronic patient record<br>• Multiple meetings with data analysts Business Intelligence (BI) to visualize population data output in dashboard<br>• One patient representatives has been engaged as an employee during the VBHC implementation *<br><br>**Deliverables**:<br>• Registration form for clinical outcomes and case mix variables<br>• Embedded PROM tool including dashboard for the involved healthcare professionals	**Activities**:<br>• Team is trained how to use the clinical registration forms<br>• Team is trained how to use the PROM tool, patients are invited to fill in the PROMs<br><br>**Deliverables**:<br>• Insight PROMs on patient-level which are discussed in team before consultation<br>• Insight population data in BI dashboard
	Facilitators	• Enthusiastic team including 2 patients was created by doing these workshops together (including speed dates), this created a positive team feeling.<br>• Department invested in available hours to facilitate workshops during work hours.<br>• The workshops were facilitated by the clinical lead along with the consultant. During the workshops team member also worked in subgroups. This both provided ownership of the team.<br>• Creating a common 'dream' was an inspiring experience.<br>• Visualization of the care pathway based on patients' experiences brought many insights and points for learning and improvement. An additional advantage was that this could also be a starting point for the PROMs by gaining insight into the impact for patients during the care process.<br>• Inspiration from good examples in other hospitals<br>• Focus group clearly indicated what the patients need. This was clearly different from the thoughts of the healthcare professionals.		• The clinical lead as driving force was representative, there was acknowledgement by the team)<br>• Exposure of the outpatient CKD clinic by sharing knowledge during several presentations in the Netherlands.<br>• Easy accessible cooperation and contact among team members<br>• Support (in time and knowledge) from the supporting departments has been essential for success	
	Challenges	• There was a lot of commitment of the department, but it was hard to find people from other disciplines to participate in our value team.<br>• It was quite challenging to find a representative group of patients for the focus groups. As is often the case, mainly the highly educated and eager patients joined these sessions.		• Pioneer function takes time and frustration<br>• Choice by steering committee and board of directors for the tool selection for patient-reported outcome measurement took a long time, this caused a lot of delay.<br>• Lateralization (and merger) of the value team to a 'new' location causes delay and limited implementation.<br>• It was challenging to get a representative patient population dashboard with data output of the EHR. In addition, there was a lack of capacity to create a dashboard for the 'new' location on short notice after lateralization.	
